# Sodium Glycerophosphate vs. Inorganic Phosphate Use in Parenteral Nutrition for Preterm Infants: A Retrospective Study

**DOI:** 10.3390/children12020229

**Published:** 2025-02-13

**Authors:** Jung-Ting Chang, Yu-Jun Chang, Lih-Ju Chen, Cheng-Han Lee, Hsiao-Neng Chen, Jia-Yuh Chen, Chien-Chou Hsiao

**Affiliations:** 1 Department of Neonatology, Changhua Christian Children’s Hospital, No. 320, Xuguang Rd., Changhua City 500010, Taiwan; b101098104@tmu.edu.tw (J.-T.C.); 168706@cch.org.tw (L.-J.C.); 129130@cch.org.tw (C.-H.L.); 19184@cch.org.tw (H.-N.C.); 182288@cch.org.tw (J.-Y.C.); 2 Big Data Center, Changhua Christian Hospital, No. 135, Nanxiao St., Changhua City 500209, Taiwan; 83686@cch.org.tw; 3 Department of Post-Baccalaureate Medicine, College of Medicine, National Chung Hsing University, No. 145, Xingda Rd., South Dist., Taichung City 402202, Taiwan

**Keywords:** sodium glycerophosphate, inorganic phosphate, parenteral nutrition, infant, premature

## Abstract

Background/Objectives: Sodium glycerophosphate improves the adverse side effects of parenteral nutrition. Therefore, this study aimed to evaluate different outcomes, including metabolic bone disease and electrolyte imbalance, associated with the use of sodium glycerophosphate or inorganic phosphate in parenteral nutrition for preterm neonates. Methods: This retrospective cohort study enrolled 402 newborns admitted to the neonatal intensive care unit of one medical center between January 2019 and September 2021. Of them, 205 received sodium glycerophosphate as parenteral nutrition, while the other 197 received inorganic phosphate. Baseline characteristics and growth parameters, including body weight, body length, and head circumference in the first year of life; calcium and phosphate content of parenteral nutrition in the first 4 weeks; calcium, phosphorus, alkaline phosphatase (ALP), and creatinine levels; and morbidities were compared. Results: During the first 4 weeks, the calcium and phosphate contents of parenteral nutrition were significantly higher in the sodium glycerophosphate vs. inorganic phosphate group. Growth parameters did not differ significantly between groups. The sodium glycerophosphate group showed a higher mean serum phosphate level (4.0 ± 1.2 mg/dL vs. 3.5 ± 1.3 mg/dL, *p* = 0.001), lower serum ALP level (402.8 ± 202.8 U/L vs. 466.4 ± 228.6 U/L, *p* = 0.004), lower seizure incidence (4.9% vs. 13.2%, *p* = 0.003), and higher hypocalcemia incidence (41.5% vs. 31.5%, *p* = 0.038). However, there were no significant intergroup differences in other common morbidities such as metabolic bone diseases of prematurity, bronchopulmonary dysplasia, electrolyte imbalance, hypoglycemia, retinopathy of prematurity, or intraventricular hemorrhage. Conclusions: Compared to inorganic phosphate, sodium glycerophosphate is associated with higher serum phosphate levels, lower ALP levels, and reduced seizure incidence in premature infants. However, as the study was retrospective and single-center, further randomized controlled trials are needed to confirm these findings.

## 1. Introduction

The greatest mineral accretion in the fetus occurs during the last trimester of pregnancy [1]. Therefore, premature birth and low birth weight are strongly associated with metabolic bone diseases, including delayed longitudinal growth, osteopenia, and rickets [1,2,3,4]. If very low birth weight infants cannot maintain appropriate bone accretion due to inadequate calcium and phosphate levels, premature metabolic bone disease may develop, leading to osteomalacia, long bone fractures, and impaired linear growth [5,6]. Although complete enteral feeding can prevent these problems, most infants cannot tolerate enteral feeding within the first few days or weeks of life. Therefore, nutritional supplementation with elevated calcium and phosphate concentrations is usually administered parenterally to prevent metabolic bone diseases or electrolyte imbalances. Consequently, the administration of calcium and phosphate via parenteral nutrition is important.

Inorganic phosphate has recently been used as a phosphate source in parenteral nutrition. In growing preterm infants, the updated European Society of Pediatric Gastroenterology, Hepatology, and Nutrition guidelines recommend the parenteral nutrition administration of 1.6–3.5 mmol/kg/day (64–140 mg/kg/day) of calcium and 1.6–3.5 mmol/kg/day (50–108 mg/kg/day) of phosphate, with an approximate calcium-to-phosphate molar ratio of 1.3 (approximate mass ratio, 1.7) [5,7]. However, the risk of precipitation associated with an imbalanced calcium-to-phosphate ratio may lead to insufficient calcium and phosphate concentrations in preterm neonates. Sodium glycerophosphate was recently found to improve the precipitation of parenteral nutrition. Compared with inorganic salts, organic calcium and phosphate in parenteral nutrition solutions have higher compatibility and lower precipitation risks [5,8]. The use of sodium glycerophosphate allows greater concentrations of calcium and phosphorus to be administered via parenteral nutrition, as either phosphate from infused glycerophosphate is directly available or phosphorus becomes displaced from the intracellular pool [9]. Therefore, parenteral nutrition solutions containing sodium glycerophosphate provide a relatively high mineral content. Conversely, the high sodium content of sodium glycerophosphate limits mineral intake in parenteral nutrition regimens [10,11,12].

Nonetheless, few studies have discussed the short-term outcomes of sodium glycerophosphate use in parenteral nutrition, including metabolic bone diseases of prematurity and long-term growth after discharge, including body weight, length, and head circumference, in the first year of life. Hence, here we aimed to investigate the differences in neonatal outcomes between the use of sodium glycerophosphate and inorganic phosphate for parenteral nutrition at our center in recent years.

## 2. Materials and Methods

This retrospective cohort study enrolled all preterm infants with a gestational age of less than 37 weeks who were admitted to the neonatal intensive care unit of Changhua Christian Children’s Hospital and received parenteral nutrition for at least 7 days between January 2019 and September 2021. The exclusion criteria were incomplete data or loss of follow-up after discharge, gastrointestinal tract malformations requiring surgery, complex congenital heart disease, chromosomal abnormalities, or death before discharge. The patients were divided according to the administered parenteral nutrition solution. As sodium glycerophosphate was introduced into parenteral nutrition regimens in May 2020 to replace inorganic phosphate, patients born between January 2019 and May 2020 were included in the inorganic phosphate group, while those born between May 2020 and September 2021 were included in the sodium glycerophosphate group. Patients who were administered both inorganic phosphate and sodium glycerophosphate were excluded. Since May 2020, sodium glycerophosphate has been used as a first-line additive for parenteral nutrition. The compositions of the parenteral nutrition solutions are listed in Supplementary Table S1.

Throughout the study period, parenteral nutrition was prescribed based on the recommendations of the Taiwan Society of Neonatology, which states a lower limit of the recommended range of 64 mg/kg/day of calcium, 5 mg/kg/day of magnesium, and 50 mg/kg/day of phosphorus. The initial target dose of phosphorus was 31 mg/kg/day for preterm infants [13]. We applied the same nutritional care strategy to all enrolled infants during the relevant periods, including fluid intake, mineral supplementation, and feeding plans, with the exception that organic phosphate replaced the parenteral nutrition formulas. Postnatally, a parenteral nutritional solution containing inorganic phosphate or sodium glycerophosphate is administered as soon as possible. Upon reaching a feeding volume of approximately 100 mL/kg/day, parenteral nutrition was discontinued, and fortification with human milk was spontaneously initiated, while the goal for full enteral feeding volume was 140–150 mL/kg/day. Vitamin D supplementation using LiquiD P&B (U-LONG PHARMACEUTICAL CO., LTD.; Taipei, Taiwan) was initiated in preterm infants once they tolerated breastfeeding. If severe hypercalcemia or hyperphosphatemia occurred, the calcium or phosphate supplementation was temporarily discontinued. Conversely, the possibility of hypocalcemia-induced hypophosphatemia, or vice versa, was considered and appropriately addressed.

The patients’ baseline characteristics included birth history (gestational age, birth weight, sex, and maternal history), growth parameters (Z-score) at different ages from birth until 1 year of age (body weight, body length, head circumference, and growth difference), feeding or nutritional conditions (calcium and phosphate content of parenteral nutrition in the first 4 weeks), laboratory results during hospitalization (lowest serum calcium and phosphorous levels and highest alkaline phosphatase [ALP] and creatinine levels), and morbidities.

Growth parameters were adjusted with the Z-score using the calculator tools INTERGROWTH-21st and WHO Growth Standard for 0–24 months [14]. Metabolic bone diseases of prematurity were diagnosed by bone rarefaction associated with metaphyseal alterations or subperiosteal bone formations associated with the presence of spontaneous fractures with radiological evidence but no use of serum biochemical markers [15]. Acute kidney injury (AKI) was defined as an elevated serum creatinine level (increase to 1.5× baseline within the prior 7 days, or increase of ≥0.3 mg/dL within 48 h) or decreased urine volume (<0.5 mL/kg/h for >6 h) according to the Kidney Disease Improving Global Outcomes criteria for AKI in children [16]. Bronchopulmonary dysplasia was diagnosed based on the need for oxygen supplementation and/or positive-pressure support at a postmenstrual age of 36 weeks or at discharge according to the 2001 National Institute of Child Health and Human Development consensus workshop criteria [17]. Seizures were defined based on the clinicians’ high degree of suspicion, informed by clinical history, or according to the presence of encephalopathy with or without abnormal paroxysmal movements. These movements were evaluated using electroencephalography, and the epileptogenic activity was confirmed by a pediatric neurologist. Meningitis was defined as cerebrospinal fluid with abnormal values or positive culture. An ophthalmologist diagnosed cases of retinopathy of prematurity and classified their severity. Necrotizing enterocolitis was defined as stage IIa or higher according to Bell criteria. Intraventricular hemorrhage was diagnosed and graded using brain ultrasonography according to the Papile classification [18]. The criteria for electrolyte imbalance included hypernatremia (serum sodium > 145 mmol/L), mild hyponatremia (130 mmol/L < serum sodium < 135 mmol/L), moderate hyponatremia (serum sodium < 130 mmol/L), severe hyponatremia (serum sodium < 120 mmol/L) [19], hyperkalemia (serum potassium > 6 mmol/L), hypokalemia (serum potassium < 3.5 mmol/L) [20], hypocalcemia (serum calcium < 7 mg/dL, 1.75 mmol/L), and hypophosphatemia (serum phosphorus < 5 mg/dL, 1.6 mmol/L) [7]. Blood samples were collected weekly according to standard routines until the parenteral nutrition was discontinued.

Continuous variables are presented as median and interquartile range (25–75th percentiles), whereas categorical variables are presented as numbers and percentages. As most continuous variables had positively skewed distributions, the Mann–Whitney U test was used to compare the median values between groups, whereas the chi-squared or Fisher’s exact test was used to compare categorical variables. Bivariate and multivariate logistic regression analyses were performed to assess the association between sodium glycerophosphate use and seizures. All data were analyzed using IBM SPSS Statistics for Windows (version 22.0; IBM Corp., Armonk, NY, USA). Statistical significance was set at *p* < 0.05.

This study was conducted in accordance with the principles of the Declaration of Helsinki and approved by the Research Ethics Board Committee of Changhua Christian Children’s Hospital (CCH IRB no. 220523; approval date: 22 January 2022), which waived the requirement for informed consent due to the retrospective nature of the study.

## 3. Results

**Figure 1:**
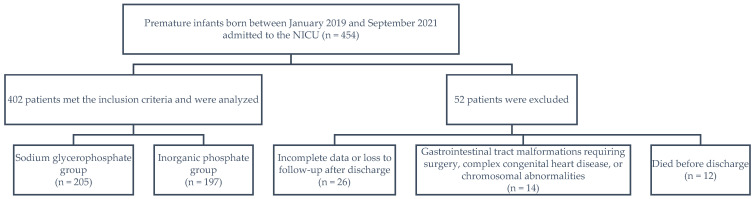
Patient inclusion flowchart. NICU, neonatal intensive care unit.

A total of 454 preterm neonates born between January 2019 and September 2021 were eligible. Of them, 52 patients were excluded, including 26 for whom data were incomplete or who were lost to follow-up after discharge; 14 who had gastrointestinal tract malformations requiring surgery, complex congenital heart disease, or chromosomal abnormalities; and 12 who died before discharge. Finally, 402 preterm infants were included, 205 in the sodium glycerophosphate group and 197 in the inorganic phosphate group (Figure 1).

There were no significant intergroup differences in baseline maternal characteristics in terms of preeclampsia, intrauterine growth restriction, and antenatal corticosteroid use; however, the incidence of maternal gestational diabetes mellitus (19.0% vs. 4.1%; *p* < 0.001) and pregnancy-induced hypertension (14.6% vs. 4.1%; *p* = 0.003) was higher in the sodium glycerophosphate vs. inorganic phosphate group tip (Table 1). Additionally, neonatal characteristics including gestational age, sex, birth weight, feeding type, parenteral nutrition duration, time to full feeding, length of hospital stay, and diuretic use did not differ significantly between groups. Nevertheless, patients in the sodium glycerophosphate group received vitamin D supplementation more frequently than those in the inorganic phosphate group (94.6% vs. 85.3%; *p* = 0.002).

Calcium and phosphate contents in the parenteral nutrition group were significantly higher in the sodium glycerophosphate vs. inorganic phosphate group at all time points (Table 2). The average calcium and phosphate additive levels were higher and more stable in the sodium glycerophosphate vs. the inorganic phosphate group. Nevertheless, the recommended ranges for growing preterm infants, 64–140 mg/kg/day for calcium and 50–108 mg/kg/day for phosphorus [7], had not yet been achieved. The enteral mineral intakes of calcium and phosphate were 120–140 and 60–90 mg/kg/day, respectively [21].

The sodium glycerophosphate group exhibited a lower calcium-to-phosphate ratio than the inorganic phosphate group. In early parenteral nutrition, when calcium and phosphorus intakes are low and protein and energy management is optimized, a calcium-to-phosphate molar ratio below 1 (0.8–1.0) is recommended to reduce the incidence of early postnatal hypercalcemia and hypophosphatemia [5,7,22].

The lowest serum phosphate levels during hospitalization in the sodium glycerophosphate group were above those of the inorganic phosphate group (serum phosphorus: 4.0 ± 1.2 mg/dL vs. 3.5 ± 1.3 mg/dL, *p* = 0.001) (Table 3), with no significant intergroup difference in hypophosphatemia incidence (Table 4). The groups did not differ significantly in the lowest serum calcium levels during hospitalization; however, the sodium glycerophosphate group had a higher incidence of hypocalcemia (41.5% vs. 31.5%, *p* = 0.038). No significant differences were observed in other electrolyte imbalances, including hypernatremia, hyponatremia, hyperkalemia, and hypokalemia. The highest serum ALP levels during hospitalization were higher in the inorganic phosphate vs. sodium glycerophosphate group (466.4 ± 228.6 U/L vs. 402.8 ± 202.8 U/L, *p* = 0.004). Additionally, a greater percentage of infants in the inorganic phosphate group had serum ALP levels exceeding 500 U/L compared to those in the sodium glycerophosphate group (37.6% vs. 24.6%, *p* = 0.007). Simultaneously, the incidence of metabolic bone diseases in premature infants did not differ between groups, nor did the highest serum creatinine levels or incidence of AKI.

Growth parameters (Z-scores) at different time points until 1 year of age, including body weight, body length, head circumference, and growth difference at 1 year, showed no significant intergroup differences. Moreover, other common morbidities, including metabolic bone diseases of prematurity, bronchopulmonary dysplasia, hypoglycemia, sepsis, necrotizing enterocolitis, retinopathy of prematurity, and intraventricular hemorrhage, did not differ significantly between groups. No serious adverse events were observed. Surprisingly, the seizure incidence was significantly lower in the sodium glycerophosphate vs. the inorganic phosphate group (4.9% vs. 13.2%, *p* = 0.003). Since seizures are associated with ischemic stroke, intracranial hemorrhage, subdural hemorrhage, intraventricular hemorrhage, meningitis, brain malformation, genetic syndrome, electrolyte imbalance, hyperglycemia or hypoglycemia, and drug use (Supplementary Table S2) [23], independent variables were screened to determine seizure risk factors and avoid collinearity in the multivariable model. Both bivariable (odds ratio, 2.965; 95% confidence interval [CI], 1.390–6.325) and multivariable (odds ratio, 3.073; 95% CI, 1.254–7.527) analyses demonstrated an association between sodium glycerophosphate use and a reduced risk of seizures (Supplementary Table S3).

## 4. Discussion

A high prevalence of early hypophosphatemia and concomitant hypercalcemia has been observed in early and overly nourished preterm infants, which is possibly correlated with refeeding syndrome and an inadequate phosphorus supply [24]. Our preliminary results demonstrated that the introduction of sodium glycerophosphate in parenteral nutrition promoted higher mineral levels in preterm neonates and was associated with a lower seizure incidence and serum ALP levels during hospitalization. Parenteral supplementation with sodium glycerophosphate provides organic calcium and phosphate solutions with higher compatibility and a lower precipitation risk. Furthermore, with proper education and training on its use, prescription practices for sodium glycerophosphate are expected to improve, allowing for higher levels of calcium and phosphate to be incorporated into parenteral nutrition. An early supply of organic phosphorus reportedly results in higher mean serum phosphorus levels on days 1–3 and 7 [25], a finding that is consistent with the results of our study.

Previous studies reporting several nutritional strategies expected organic phosphate to provide adequate mineral nutrition for preterm infants [10,26]. Parenteral nutrition with intravenous glycophosphorus administered to very low birth weight infants resulted in low serum ALP levels and high bone mineral content but had no association with serum calcium, phosphorus, or vitamin D levels [26]. Furthermore, the administration of sodium glycerophosphate in parenteral nutrition to extremely low birth weight infants resulted in high serum calcium and phosphorus levels after day 14 and low ALP levels after day 56 [10], whereas the administration of sodium glycerophosphate promoted significantly higher serum phosphorus levels with no obvious effect on serum calcium levels. Therefore, sodium glycerophosphate may be an advantageous mineral supplement for high-risk infants.

Although we found no association between metabolic bone diseases of prematurity and either treatment in this study, we believe that the use of sodium glycerophosphate has a promising role in decreasing their incidence. Our diagnostic criteria for metabolic bone diseases of prematurity considered only radiological examinations and excluded serum biochemical markers. However, the absence of significant demineralization or fractures makes radiography unreliable in the early stages of bone disease [15,27]. ALP is the most widely accepted specific biochemical marker of metabolic bone disease during prematurity [28]. In fact, an ALP level > 500 IU/L suggests impaired bone homeostasis, while a level > 700 IU/L is associated with bone demineralization despite the absence of clinical signs [16,29]. Figueroas-Aloy et al. established a diagnosis of metabolic bone disease of prematurity with an ideal cut-off serum ALP level of 500 IU/L [30]. In the present study, the use of sodium glycerophosphate significantly lowered serum ALP levels, suggesting that it may reduce the risk of metabolic bone diseases in premature infants. In the present study, the use of sodium glycerophosphate significantly lowered serum ALP levels and resulted in a lower percentage of infants with the highest serum ALP > 500 U/L, suggesting that it may reduce the risk of metabolic bone disease in premature infants.

A previous study reported no differences in body weight, body length, or head circumference in extremely low birth weight infants with the use of sodium glycerophosphate; however, the study analyzed only infants with a postmenstrual age of 36 weeks and corrected age of 6 months [10]. We included more participants and investigated later growth parameters until 1 year of age; our results indicated no differences in body weight, length, or head circumference Z-scores during that time. The early amino acid intake of premature infants is associated with enhanced endogenous insulin production as well as phosphate and potassium transfer into cells for energy production along with glycogen, fat, and protein synthesis [31]. Therefore, higher calcium and phosphate doses may not induce bone mineral acceleration or longitudinal growth.

Our study also found a significantly higher incidence of hypocalcemia in the sodium glycerophosphate group. We attribute this to the fact that the calcium and phosphate concentrations provided by sodium glycerophosphate did not reach the recommended range for preterm infants. Moreover, the higher incidence of maternal gestational diabetes mellitus and pregnancy-induced hypertension in the sodium glycerophosphate group may have contributed to the risk of neonatal hypocalcemia resulting from maternal hypocalcemia [32,33]. However, further studies are required to clarify the association between sodium glycerophosphate levels and neonatal hypocalcemia.

Our study demonstrated a lower incidence of seizures in the sodium glycerophosphate group, which is possibly associated with the significantly higher ratio of vitamin D supplementation. Higher levels of vitamin D supplementation may reflect increased awareness of its importance in clinical practice, aligning with the evolving standards of modern medical care. Neonates with vitamin D deficiency may be at high risk of developing hypocalcemic seizures, especially those with maternal vitamin D deficiency [34,35]. Exclusively breastfed infants with hypocalcemic seizures have low serum calcifediol and elevated parathyroid hormone (PTH) levels [36]. Severe hypophosphatemia secondary to phosphate-deficient hyperalimentation causes nervous system abnormalities, and signs suggestive of metabolic encephalopathy (e.g., irritability, paresthesia, seizures, and coma) have been observed in adolescents and adults [37,38]. Hypophosphatemia may cause serious muscular, neurological, and hematologic disorders as well as peripheral neuropathy with paresthesia and metabolic encephalopathy, a disorder marked by confusion, seizures, and coma [39,40]. Therefore, hypophosphatemia should also be considered a cause of seizures in addition to hypernatremia, hypocalcemia, and hypomagnesemia.

Our study had several limitations. First, the observational design of a single retrospective cohort made the data less comprehensive and precluded the establishment of causality. We used data from the medical records to increase the sample size and improve the accuracy and reliability of the results. Second, our definition of metabolic bone disease at prematurity excluded biochemical markers that omitted early demineralization of bone disease. Further studies should assess the biochemical markers of bone metabolism, including vitamin D and intact PTH levels, and use dual-energy X-ray absorptiometry or quantitative ultrasound to evaluate bone quality, which can help screen for metabolic bone diseases in the asymptomatic phase [15]. Finally, although several side effects of sodium glycerophosphate have been reported, renal calculus formation remains a concern; however, renal ultrasonography is not routinely performed to evaluate renal calculi. Despite these limitations, our findings have significant implications for preterm infants administered sodium glycerophosphate in parenteral nutrition to achieve higher calcium and phosphate concentrations, which may be associated with lower serum ALP levels and neonatal seizure incidence.

## 5. Conclusions

Sodium glycerophosphate is a beneficial option for infant nutrition, as its use in parenteral nutrition helps maintain optimal calcium and phosphate levels until full enteral feeding is achieved. Compared to inorganic phosphate, its use in premature infants is associated with higher serum phosphate levels, lower ALP levels, and reduced seizure incidence. However, as the study was retrospective and single-center, further randomized controlled trials are needed to confirm these findings.

## Figures and Tables

**Table 1:** Baseline characteristics by study group.

		Inorganic Phosphate (*n* = 197)		Sodium Glycerophosphate (*n* = 205)		
		N	%	N	%	*p*-Value
**Maternal factor**						
GDM		8	4.1	39	19.0	<0.001
PIH		8	4.1	30	14.6	0.003
Preeclampsia		41	20.8	55	26.8	0.157
IUGR		21	10.7	28	13.7	0.358
Antenatal corticosteroid use		143	72.6	165	80.5	0.061
**Neonatal factor**						
Sex	Male	92	46.7	110	53.7	0.163
	Female	105	53.3	95	46.3	
BW at birth (g)	<1000	50	25.4	58	28.3	0.687
	1000–1499	76	38.6	81	39.5	
	>1500	71	36.0	66	32.2	
Feeding type	Breast milk	92	46.7	110	53.7	0.315
	Premature formula milk	53	26.9	52	25.3	
	Mixed	52	26.4	43	21.0	
Feeding with vitamin D		168	85.3	194	94.6	0.002
Diuretic use		43	21.8	49	23.9	0.621

GDM, gestational diabetes mellitus; IUGR, intrauterine growth restriction; PIH, pregnancy-induced hypertension. *p*-values determined by chi-squared test or Fisher’s exact test as appropriate.

**Table 2:** Parenteral intakes of the sodium glycerophosphate and inorganic phosphate groups.

Mean (SD)	Inorganic Phosphate (*n* = 197)	Sodium Glycerophosphate (*n* = 205)	*p*-Value
Gestational age (weeks)	30.5 (3.2)	30.2 (3.1)	0.304
Time to full feeding (days)	18.4 (12.2)	19.3 (11.3)	0.095
PN duration (days)	19.0 (13.4)	20.0 (15.8)	0.347
Length of hospital stay (days)	58.7 (35.8)	63.5 (40.4)	0.307
Average calcium per week (mg/kg/day)			
Week 1	228.5 (53.2)	340.6 (83.8)	<0.001
Week 2	172.4 (98.9)	351.8 (144.0)	<0.001
Week 3	101.2 (108.1)	249.0 (207.0)	<0.001
Week 4	55.3 (93.5)	128.9 (192.8)	0.014
Average phosphate per week (mg/kg/day)			
Week 1	12.8 (3.3)	23.0 (6.6)	<0.001
Week 2	12.4 (8.8)	27.5 (11.4)	<0.001
Week 3	7.1 (8.2)	20.2 (17.0)	<0.001
Week 4	3.9 (7.2)	11.2 (16.6)	0.011
Average Ca/P ratio per week			
Week 1	1.3 (0.3)	1.2 (0.2)	<0.001
Week 2	1.0 (0.5)	0.9 (0.3)	<0.001
Week 3	0.7 (0.6)	0.6 (0.5)	0.007
Week 4	0.4 (0.6)	0.3 (0.4)	0.315

PN, parenteral nutrition; Ca/P ratio, calcium-to-phosphorus ratio; SD, standard deviation. *p*-values determined by Mann–Whitney U test.

**Table 3:** Growth and laboratory outcomes by study group.

Mean (SD)	Inorganic Phosphate (*n* = 197)	Sodium Glycerophosphate (*n* = 205)	*p*-Value
At birth (Z-score)			
BW	−0.54 (1.15)	−0.50 (1.09)	0.720
BL	−0.82 (1.24)	−0.79 (1.20)	0.585
HC	−0.70 (1.10)	−0.69 (1.12)	0.896
At 1 month old (Z-score)			
BW	−0.86 (1.39)	−0.77 (1.35)	0.258
BL	−1.38 (1.68)	−1.40 (1.68)	0.735
HC	−0.87 (1.57)	−0.97 (1.57)	0.541
At 2 months old (Z-score)			
BW	−0.28 (1.44)	−0.17 (1.49)	0.477
BL	−1.06 (1.88)	−1.17 (1.93)	0.586
HC	−0.10 (1.76)	−0.34 (1.77)	0.281
At 6 months old (Z-score)			
BW	−0.37 (1.29)	−0.19 (1.35)	0.061
BL	−0.67 (1.42)	−0.45 (1.41)	0.115
HC	−0.23 (1.33)	−0.16 (1.34)	0.472
At 1 year old (Z-score)			
BW	−0.48 (1.25)	−0.40 (1.29)	0.440
BL	−0.61 (1.32)	−0.39 (1.34)	0.104
HC	−0.41 (1.27)	−0.38 (1.32)	0.823
Growth difference at 1 year (Z-score)			
BW	0.06 (1.39)	0.10 (1.25)	0.804
BL	0.20 (1.45)	0.40 (1.35)	0.139
HC	0.29 (1.45)	0.30 (1.45)	0.921
Serum calcium (mg/dL) *	7.4 (1.1)	7.3 (1.1)	0.419
Serum phosphate (mg/dL) †	3.5 (1.3)	4.0 (1.2)	0.001
Serum ALP (U/L) ‡	466.4 (228.6)	402.8 (202.8)	0.004
Serum creatinine (mg/dL) §	1.1 (0.8)	1.1 (0.6)	0.337

* Lowest serum calcium levels during hospitalization. † lowest serum phosphate levels during hospitalization. ‡ highest serum ALP levels during hospitalization. § highest serum creatinine levels during hospitalization. ALP, alkaline phosphatase; BL, body length; BW, body weight; HC, head circumference; SD, standard deviation. *p*-values determined by the Mann–Whitney U test.

**Table 4:** Laboratory outcomes and comorbidities in the sodium glycerophosphate and inorganic phosphate groups.

		Inorganic Phosphate (*n* = 197)		Sodium Glycerophosphate (*n* = 205)		
		N	%	N	%	*p*-Value
Serum ALP > 500 *		71	37.6	50	24.6	0.007
Hypocalcemia †		62	31.5	85	41.5	0.038
Hypophosphatemia ‡		165	84.2	160	78.0	0.117
Hypernatremia		33	16.8	29	14.1	0.470
Hyponatremia	Mild	102	51.8	108	52.7	0.955
	Moderate	66	33.5	64	31.2	
	Severe	4	2.0	5	2.4	
Hyper-/hypokalemia	Hyperkalemia	8	4.1	3	1.5	0.371
	Hypokalemia	78	39.6	90	43.9	
	Both	18	9.1	16	7.8	
Retinopathy of prematurity	Stage 1	30	15.2	20	9.8	0.422
	Stage 2	12	6.1	13	6.3	
	Stage 3	13	6.6	12	5.9	
	Stage 4	0	0.0	1	0.5	
Bronchopulmonary dysplasia		42	21.3	44	21.5	0.972
Acute kidney injury		33	16.8	25	12.2	0.194
Metabolic bone diseases		17	8.6	11	5.4	0.199
Sepsis		27	13.7	37	18.0	0.234

* Highest serum ALP > 500 U/L. † hypocalcemia (serum calcium < 7 mg/dL, 1.75 mmol/L). ‡ hypophosphatemia (serum phosphorus < 5 mg/dL, 1.6 mmol/L).

## Data Availability

All data relevant to this study have been included in the article or uploaded as Supplementary Information.

## Supplementary Materials

The following supporting information can be downloaded at https://www.mdpi.com/article/10.3390/children12020229/s1, Table S1: Electrolyte and Lyo-Povigent composition of parenteral nutrition; Table S2: Etiology of neonatal seizure among infants using Sodium glycerophosphate; Table S3: Logistic regression analysis of seizure.
