# Supplementary material for: Sodium Glycerophosphate vs. Inorganic Phosphate Use in Parenteral Nutrition for Preterm Infants: A Retrospective Study
Source: Children (Basel). 2025 Feb 13;12(2):229. doi: 10.3390/children12020229 (PMC11853877; doi:10.3390/children12020229)
Supplement: Supplementary file 1 [file children-12-00229-s001.zip › children-3439598-supplementary.pdf]

Supplementary Tables

Supplementary Table S1. Electrolyte and Lyo-Povigent composition of parenteral nutrition

Ingredients	Pharmaceutical Preparation	Composition Unit
	Electrolytes	
Sodium	Sodium chloride 3%	0.5 mmol//mL
	Sodium glycerophosphate	2 mmol//mL
Potassium	Potassium chloride 15%	2 mmol/mL
	Potassium phosphate	4.4 mmol/mL
Calcium	Calcium gluconate 10%	0.2325 mmol/mL
Phosphate	Sodium glycerophosphate	1 mmol/mL
	Potassium phosphate	3 mmol/mL
Magnesium	Magnesium sulfate 10%	0.4 mmol/mL
	Lyo-Povigent (4 mL/vial)	
Vitamin D	Ergocalciferol	200 IU

The proportion of parenteral nutrition would be admixed to cover the needs of individual neonates

Supplementary Table S2. Etiology of neonatal seizure among infants using Sodium glycerophosphate

		**Inorganic Phosphate (n = 197)**		**Sodium Glycerophosphate (n = 205)**		
		N	%	*N*	%	***p*-Value**
Ischemic stroke		0	0.0	0	0.0	
Intracranial hemorrhage		1	0.5	0	0.0	0.621
SDH		1	0.5	3	1.5	0.623
Meningitis		1	0.5	1	0.5	>0.999
Hydrocephalus		4	2.0	3	1.5	0.719
Brain malformation		0	0.0	1	0.5	>0.999
Genetic syndrome*		0	0.0	0	0.0	
Hyper/Hypoglycemia	Hyper	40	20.3	56	27.3	0.234
	Hypo	43	21.8	50	24.4	
	Both	26	13.2	25	12.2	

HIE: Hypoxic-ischemic encephalopathy; SDH: Subdural hemorrhage; *Genetic syndrome (including benign familial neonatal epilepsy)

P-value by chi-square test or Fisher's exact test when appropriate

Supplementary Table S3. Logistic regression analysis of seizure

			Seizure		Bivariable Analysis (Crude)			Multivariable Analysis (Adjusted)		
**Variable**		*Total*	*N*	*%*	*Odds Ratio*	*95% CI*	*p-Value*	*Odds Ratio*	*95% CI*	*p-Value*
Full feeding duration (days)	Median (IQR)	16.0 (11.0–23.0)	32.0 (19.0–42.0)		1.094	1.060–1.128	<0.001	1.080	1.046–1.115	<0.001
Ca/P ratio per week	Median (IQR)	1.2 (1.1–1.4)	1.4 (1.2–1.7)		15.390	4.797–49.379	<0.001	5.841	1.490–22.898	0.011
Sodium Glycerophosphate use	No	197	26	13.2	2.965	1.390–6.325	0.005	3.073	1.254–7.527	0.014
	Yes	205	10	4.9	1.000			1.000		
Antenatal corticosteroid use	No	94	11	11.7	1.500	0.709–3.177	0.289			
	Yes	308	25	8.1	1.000					
Diuretic use	No	310	18	5.8	1.000					
	Yes	92	18	19.6	3.946	1.957–7.957	<0.001			
Feeding with vitamin D	No	40	5	12.5	1.525	0.557–4.174	0.411			
	Yes	362	31	8.6	1.000					
Intraventricular hemorrhage	No	357	25	7.0	1.000			1.000		
	Yes	45	11	24.4	4.296	1.946–9.487	<0.001	3.199	1.241–8.248	0.016
Hydrocephalus	No	395	32	8.1	1.000					
	Yes	7	4	57.1	15.125	3.243–70.549	0.001			
Hypernatremia	No	340	21	6.2	1.000					
	Yes	62	15	24.2	4.848	2.336–10.059	<0.001			
Hyponatremia	Nil	53	4	7.5	1.000					
	Mild	210	13	6.2	0.808	0.253-2.588	0.720			
	Moderate	130	16	12.3	1.719	0.547-5.406	0.354			
	Severe	9	3	33.3	6.125	1.096-34.216	0.039			
Hypocalcemia	No	255	15	5.9	1.000					
	Yes	147	21	14.3	2.667	1.328–5.353	0.006			
Hyper/Hypoglycemia	Nil	162	10	6.2	1.000		0.001			
	Hyper	96	14	14.6	2.595	1.104–6.101	0.029			
	Hypo	93	1	1.1	0.165	0.021–1.312	0.089			
	Both	51	11	21.6	4.180	1.658–10.536	0.002			

Ca/P ratio, calcium-to-phosphorus ratio; CI, confidence interval; IQR, interquartile range

P-values determined by chi-square test or Fisher's exact test when appropriated
